# Pilot study of a new freely available computer-aided polyp detection system in clinical practice

**DOI:** 10.1007/s00384-022-04178-8

**Published:** 2022-05-11

**Authors:** Thomas J. Lux, Michael Banck, Zita Saßmannshausen, Joel Troya, Adrian Krenzer, Daniel Fitting, Boban Sudarevic, Wolfram G. Zoller, Frank Puppe, Alexander Meining, Alexander Hann

**Affiliations:** 1 Interventional and Experimental Endoscopy (InExEn), Internal Medicine II, University Hospital Würzburg, Würzburg, Germany; 2 Artificial Intelligence and Knowledge Systems, Institute for Computer Science, Julius-Maximilians-Universität Würzburg, Würzburg, Germany; 3 Department of Internal Medicine and Gastroenterology, Katharinenhospital, Stuttgart, Germany

**Keywords:** Colonoscopy, Polyp, Artificial intelligence, Deep learning, CADe

## Abstract

**Purpose:**

Computer-aided polyp detection (CADe) systems for colonoscopy are already presented to increase adenoma detection rate (ADR) in randomized clinical trials. Those commercially available closed systems often do not allow for data collection and algorithm optimization, for example regarding the usage of different endoscopy processors. Here, we present the first clinical experiences of a, for research purposes publicly available, CADe system.

**Methods:**

We developed an end-to-end data acquisition and polyp detection system named EndoMind. Examiners of four centers utilizing four different endoscopy processors used EndoMind during their clinical routine. Detected polyps, ADR, time to first detection of a polyp (TFD), and system usability were evaluated (NCT05006092).

**Results:**

During 41 colonoscopies, EndoMind detected 29 of 29 adenomas in 66 of 66 polyps resulting in an ADR of 41.5%. Median TFD was 130 ms (95%-CI, 80–200 ms) while maintaining a median false positive rate of 2.2% (95%-CI, 1.7–2.8%). The four participating centers rated the system using the System Usability Scale with a median of 96.3 (95%-CI, 70–100).

**Conclusion:**

EndoMind’s ability to acquire data, detect polyps in real-time, and high usability score indicate substantial practical value for research and clinical practice. Still, clinical benefit, measured by ADR, has to be determined in a prospective randomized controlled trial.

**Supplementary information:**

The online version contains supplementary material available at 10.1007/s00384-022-04178-8.

## Introduction

Screening colonoscopies are highly effective at reducing the incidence of colorectal cancer (CRC). Previous studies revealed a decrease of 68% regarding CRC-related mortality by performing screening colonoscopies as most of these carcinomas develop over years following the adenoma-carcinoma sequence [1]. Adenoma detection rate (ADR) evolved to one of the most important colonoscopy quality parameters correlated to interval carcinoma rate [1]. As the research of artificial intelligence (AI) progressed, clinical applications were tested for viability [2]. A meta-analysis by Hassan et al. analyzed the current randomized studies regarding deep learning–based polyp detection in colonoscopy (CADe) [3]. They concluded that AI-assisted polyp detection increases the ADR, especially for small (< 5 mm), flat adenomas. Anyhow, only one of the five analyzed studies was performed in Europe [4] while the others are limited to an Asian study population [5–8]. Furthermore, three of the studies included mostly symptomatic patients [5–7]. Regarding generalizability, only one of the CADe systems [4] was evaluated with multiple processor types and only one study was multicentric [4]. Therefore, the authors concluded that more data for non-Asian populations is necessary. Furthermore, examiners focus on the center of the endoscopic image and CADe systems improve detection in the image’s periphery [9]. Lastly, to our knowledge there is no data regarding usability and acceptance of CADe systems in clinical practice.

In this study, we present the pilot phase results of our real-time CADe polyp detection system EndoMind and its framework applied in clinical practice. The proposed framework is an end-to-end solution capable of data acquisition for the training of neural networks as well as clinical application of the AI. The AI was developed utilizing multicentric data acquired by the EndoMind framework itself using different endoscopy processor types. Therefore, it is capable of fast development, evaluation, and real-time application of AI-based video analysis. Lastly, we analyzed the physicians’ feedback to evaluate the potential hardships of migrating this powerful tool for polyp detection to clinical application.

## Methods

### Development of EndoMind hardware and software

EndoMind hardware utilizes regular off-the-shelf components including a high-performance computer and a video grabber card that provide compatibility with a multitude of available endoscopy processors. The components were determined based on optimal requirements for a real-time AI application system while maintaining affordable pricing to make this freely available system easy to implement for clinicians in the future. Supplementary Table 1 lists the hardware composition resulting in a total price of about 2,880 €.

The CADe system, including software and hardware, was developed to perform data acquisition of the video signal and the exact location of the AI predictions as well as real-time polyp detection simultaneously. The software is able to handle a wide range of endoscopy processor video signals, including analog to ultra-high definition standards. The video signal is processed to single images called frames independently of the input source. Those are then forwarded to three processing pipelines (Display, AI, and Recording) in parallel to fit the requirements for real-time application (Supplementary Fig. 1). This parallelization minimizes video delay as only the predictions are visualized on a later frame. Furthermore, the AI predicts only every second to third frame and extrapolates the results to the remaining frames. The AI is based on a convolutional neural network that was trained with 506,338 manually annotated images from endoscopic examinations with and without visible polyps. The software’s detailed structure is explained in Supplementary Material. EndoMind software including a detailed installation handbook is freely available for research purposes (https://www.ukw.de/research/inexen/ai-applied-in-real-time/).

### Participants

We retrospectively reviewed colonoscopy reports and corresponding videos of our randomized controlled trial’s pilot phase data. Here, examiners with at least 10 years of experience in performing colonoscopies were asked to evaluate EndoMind before starting the randomized study phase (NCT05006092). Only complete video recordings were included. The evaluated video recordings originate from four different endoscopy processors (Olympus CV-170 and CV-190 (Olympus Europa SE & Co. KG, Hamburg, Germany), Pentax i7000 (Pentax Europe GmbH, Hamburg, Germany), and Storz TC301 (Karl Storz SE & Co. KG, Tuttlingen, Germany)). Centers included three outpatient gastroenterological practices and one community-based hospital.

### Data annotation

A physician (TJL) annotated each video from start to end and a board-certified gastroenterologist (AH) verified annotations. Sequences including polyps were labeled as such. Polyp size, morphology, pathological report if available, location and Boston bowel preparation scale (BBPS) were retrospectively identified. Polyps were categorized as proximal if located between caecum and the left flexure, otherwise as distal. Withdrawal time was determined as the time difference of the last anatomic landmark inspection (ileocecal valve, appendix, or ileum) and last image inside of the body [10]. Time spent on endoscopic interventions was manually annotated and subtracted from withdrawal time as well as all other evaluations. Each CADe prediction was labeled as true or false positive.

### Survey

Examiners of the four centers were asked to participate in an online survey about the EndoMind usage (Supplementary Table 2). The survey consisted of the System Usability Scale (SUS) resulting in a total score of 0 to 100 points. Additional questions about the EndoMind performance were rated using a Likert scale from 1 (strongly disagree) to 5 (strongly agree) or percentage estimates.

### Statistical analysis

Statistical analysis was performed using Python 3.10. Sensitivity was defined as the number of polyps detected in at least one frame divided by the number of all visible polyps. Time to first detection (TFD) was determined for each polyp as the visible time between polyp appearance and the first frame with correct CADe detection. For histology-based analyses, polyps without available histology due to not performed resection were excluded. Data was tested for normal distribution using SciPy’s normal test. For data with normal distribution, mean and standard deviation were calculated. For non-normal distributed data, median and its two-sided 95% confidence intervals (CI) were calculated using bootstrapping (*n* = 1000).

### Ethical considerations

The study was approved by the local ethical committee responsible for each study center (Ethik-Kommission Landesärztekammer Baden-Württemberg (F-2021–047), Ethik-Kommission Landesärztekammer Hessen (2021–2531), and Ethik-Kommission der Landesärztekammer Rheinland-Pfalz (2021–15,955)). All procedures were in accordance with the Helsinki Declaration of 1964 and later versions. Signed informed consent from each patient was obtained prior to participation.

## Results

### Patient characteristics

**Fig. 1:**
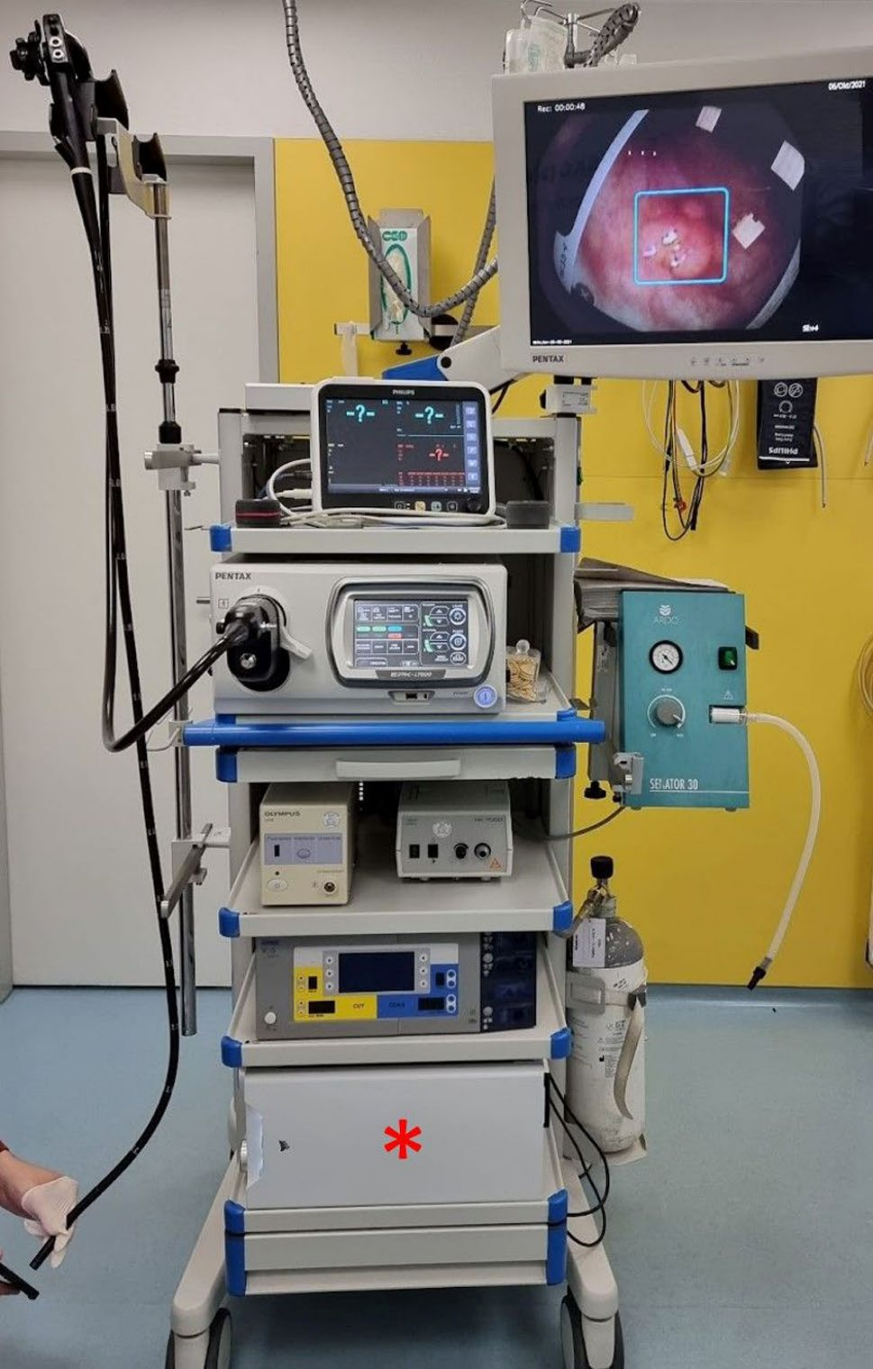
EndoMind mounted on an endoscopic tower in one of the participating centers. Presentation of a polyp image on a small screen (lower left corner) and proper detection with a bounding box (upper right corner) by EndoMind (asterisk)

Using EndoMind (Fig. 1), 41 examinations were recorded during the pilot phase of the study in four centers. Patient characteristics are presented in Table 1. Most examinations were performed for colorectal cancer screening or surveillance (63.4%). BBPS was rated as six or higher in 95.1% of the examinations. Characteristics of the participating examiners are presented in Supplementary Table 3.

**Table 1:** Patient characteristics

**Characteristic**	**Value**
Age in years, median (95% CI)	62.0 (57.0–67.0)
Gender	
Male, *n* (%)	17 (41.5)
Female, *n* (%)	24 (58.5)
Indication	
Screening or surveillance, *n* (%)	26 (63.4)
Symptomatic, *n* (%)	15 (36.6)
BBPS, median (95% CI)	7.0 (7.0–8.0)
BBPS ≥ 6, *n* (%)	39 (95.1)
BBPS < 6, *n* (%)	2 (4.9)

*CI* confidence interval, *BBPS* Boston bowel preparation scale

### CADe performance

**Fig. 2:**
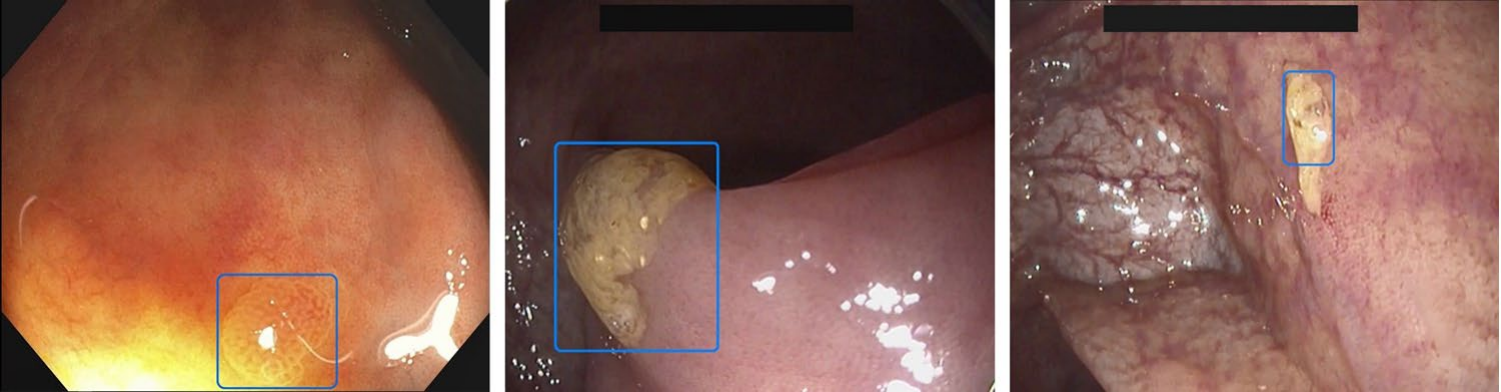
Representative selection of EndoMind detections. EndoMind correctly marks a well visible (left) and a stool covered (middle) polyp with a blue bounding box. A common cause for false positive detections represented by stool on the bowel wall is displayed in the right image

In total, 66 polyps were identified in 41 colonoscopies. Figure 2 depicts representative images of EndoMind detections. Polyp characteristics and detection metrics are summarized in Table 2. Of the 37 histologically evaluated polyps, 29 were diagnosed as adenomatous resulting in an ADR of 41.5%. EndoMind detected 29 of 29 adenomas and 66 of 66 polyps. Overall, median TFD was as fast as 130 ms (95%-CI, 80–200 ms).

**Table 2:** Polyp characteristics and CADe performance

**Category**	***n*** **(%)**	**TFD in ms, median (95%-CI)**
**All polyps**	66 (100)	130 (80–200)
**Size**		
< 5 mm	41 (62.1)	160 (80–260)
5–10 mm	19 (28.8)	120 (60–340)
> 10 mm	6 (9.1)	80 (40–4,380)
**Histology (*n* = 37)**		
Non-adenomatous	7	200 (60–2,280)
Tubular adenoma	24	160 (80–520)
Tubulovillous adenoma	3	180 (60–200)
Sessile serrated lesion	2	160 (100–220)
Carcinoma	1	40 (n.a.)
**Location**		
Proximal	30 (45.5)	160 (80–350)
Distal	36 (54.6)	120 (60–210)

*TFD* time to first polyp detection, *CI* confidence interval, *n.a.* not available

Manual annotation of all 1,544,063 individual images of which 74,422 (4.82%) contained a visible polyp, revealed an overall CADe accuracy of 95.3%. Median false positive detection rate per examination was 2.2% (95%-CI, 1.7–2.8%).

### Usability survey

Examiners participating in the pilot phase rated the usability of EndoMind with a median SUS score of 96.3 (95%-CI, 70–100). The physicians subjectively stated that 89% (95%-CI, 79–94%) of the polyps were detected by our system. Of those polyps 46% (95%-CI, 21–61%) were subjectively detected by EndoMind before the examiner. Anyhow, users partially criticized false detections as distracting (median 3, 95%-CI, 2–3) and as a possible reason for a prolonged withdrawal time (median 2.5, 95%-CI, 2.0–5.0). Lastly, interventionists agreed that the EndoMind system would benefit patient care (median 4.5, 95%-CI, 3.0–5.0) and therefore would like to use it in their clinical routine (median 4.5, 95%-CI, 4.0–5.0).

## Discussion

In this work, we present the freely available CADe system EndoMind. It incorporates recording of endoscopy videos with AI predictions. Additionally, it is capable of real-time polyp detection on a variety of endoscopy processors. We could demonstrate successful installation and use of our system in four non-research-focused centers. While previous studies included mostly symptomatic patients of Asiatic origin in a hospital setting [5–7, 11], 63.4% of the colonoscopies included in our pilot phase study were performed as screening or surveillance examinations. Furthermore, we could preliminarily validate high sensitivity (100% of polyps detected) and fast detection (median TFD 130 ms). While this preliminary data may not be directly compared to other studies, the ADR in our pilot phase study was 41.5%. A total of 29 out of 37 (78.4%) histologically evaluated polyps were diagnosed as adenoma which indicates high quality of the performed colonoscopies. Assessing the characteristics of the detected adenomas, we found a similar size distribution compared to previously published studies [4–7]. Other CAD systems report a false positive (FP) rate of 0.9 to 8% [12–14]. Assessment of false detections by EndoMind is located in the lower range with 2.2%. Qualitative screening of coherent false positive detections revealed mainly stool-covered areas, air bubbles, or pseudo-polyps generated by artifacts due to suction of the mucosa as the most common sources. As especially right-sided polyps are initially often covered by mucus, some of those FP detections may not be eliminated without severely affecting detection of these polyps in the early phase when they appear. Nevertheless, as a recent in depth analysis by Spadaccini et al. demonstrated, examiners can quickly disregard these FPs [15].

Our usability-focused survey involved only highly experienced examiners, mostly from outpatient treatment centers. We designed EndoMind to assist in screening colonoscopies; therefore, this group resembles the future target group. The participating physicians found EndoMind to be easy to use and maintain with a median SUS of 96.3 which exceeds the average of 69 [16]. Furthermore, they agreed that their clinical routine would benefit from the regular usage of EndoMind. However, the examiners also stated that false positive detections might increase their withdrawal time. Additionally, even correctly detected polyps might disturb the workflow if the physician has already identified it. Therefore, features to easily and even automatically deactivate the system should be implemented in future. While manual deactivation may be achieved by a foot switch or voice command, automatic deactivation based on the examination state seems also promising. For this, the most practical approaches include activation of the CADe system only after identification of the caecum and deactivation if an instrument is detected in the field of view. This would restrict the CADe detections to the withdrawal time and prevent disturbing activations during resections and biopsies.

Additionally, we evaluated the physician’s impressions of how many polyps were missed (11%), as well as how many polyps were detected by the system before the examiner (46%). The discrepancy between our determined sensitivity and the survey result may result from a different definition of detection: while frequently used metrics accept a polyp as detected if it is recognized at all, examiners might define a polyp, which is only detected after it is centered and focused on the image, as missed. As a more realistic measure, we therefore evaluated the TFD. Here, 89.4% of the polyps were detected in less than a second, which closely correlates with the examiners’ impression of the percentage of CADe-identified polyps.

While our results imply high clinical value of our freely available CADe system, absence of a control group in this early stage as well as the small sample size demands verification by a larger, randomized, controlled study. The aim of this study was therefore not to present how our system improves the ADR, but instead to demonstrate the application of this new CADe system in a clinical scenario involving multiple processor types and an evaluation of its performance on a frame-by-frame basis.

As our system is easy to use, and preliminary results indicate high practical value, we are confident that patient care would profit if systems like EndoMind are utilized in the daily routine. Furthermore, the implemented recording capabilities reduce the effort for continuously improving the system. By usage of rapid training iterations, our system enables for user- or patient group–specific AI fine-tuning as it is known from other applications like text to speech applications which improve their performance with increasing time of use. We hope that the EndoMind platform might contribute to improving endoscopy by continuously incorporating new AI features.

## Supplementary information

Below is the link to the electronic supplementary material.

Supplementary file1 (DOCX 300 KB)
