# Supplementary material for: Pilot study of a new freely available computer-aided polyp detection system in clinical practice
Source: Int J Colorectal Dis. 2022 May 11;37(6):1349–54. doi: 10.1007/s00384-022-04178-8 (PMC9167159; doi:10.1007/s00384-022-04178-8)
Supplement: Supplementary file 1 — Supplementary file1 (DOCX 300 KB) [file 384_2022_4178_MOESM1_ESM.docx]

**Supplementary data**

**Supplementary Material:**

*EndoMind software architecture*

The custom developed, Ubuntu based EndoMind framework runs on publicly accessible hardware. Three independent processes, further referred to as pipelines, enable real-time application without delaying the video signal.

The first step is to highlight polyps detected by the independent AI pipeline with bounding boxes. This is followed by an optional cropping step. Thereby, EndoMind can also handle 4:3 monitors with a 16:9 ratio signal from the endoscope processor. After this, the actual frame is rescaled and padded to the display output format. Finally, the remaining user interface elements (recording time, current AI version number) are drawn. Simultaneously, the independent AI pipeline is analyzing the same frame for polyps.

Since the AI is optimized for 640x640 pixel input images, the cropped frame will be resized and padded, if necessary. Afterwards, the image is uploaded to the GPU and processed by the convolutional neural network (CNN). This CNN is based on the *you only look once* (YOLO) architecture and was trained with 506,338 images from endoscopic examinations with and without polyps with manually drawn bounding boxes around polyps [1]. This results in a list of boxes including a probability-like score for each bounding box. Depending on the boxes probability score, they are forwarded to the display pipeline or disregarded.

As the AI and the display pipeline run in parallel, detected bounding boxes are displayed not on the frame on which they were detected but several frames later. The exact delay depends on the computing capabilities and the utilized model but in our case did not exceed a mean delay of 5 frames (= 100 ms at 50 frames per second). Therefore, the calculated position of a box remains close enough to highlight polyps inside the box.

The last pipeline is the recording pipeline. As there is no real-time requirement for recording, it starts with a buffer. There is only one preprocessing step, anonymization. This step optionally removes all patient information, usually printed in the black area. In this case, the frame can be cropped. However, some endoscopic setups include the patient’s name inside the camera’s field of view. In that case, the system is able to overlay the area with a black bar. The anonymized image will then be written with the video tool FFMPEG ([www.ffmpeg.org](http://www.ffmpeg.org)). This tool receives all single images and combines them to a video file stored on the hard drive disk (HDD) using a state-of-the-art encoder.

Furthermore, EndoMind is able to detect “freezes” in the live video. For this, the current frame is compared with a previous frame and, when identical, a freeze is detected by the lack of camera movement. This works well for progressive signals, but has its limitations for interlaced signals. All freezes and detections of the AI are stored in log files along with the video. The recorded videos, detection and freezes can be utilized for further evaluation.

All features are preconfigured according to the centers’ setup, and the only user control is a single button, to start and stop the AI and recording.

Besides the developed software, the Ubuntu system has been customized to initialize and function just by booting the system. After turning the computer on, the program starts without further action. For security reasons, all data-containing disks are encrypted through Linux Unified Key Setup (LUKS). This includes two HDDs. The first one is an external HDD for data exchange, it contains recorded videos and the corresponding freezes and detection files. The second one is an internal HDD to back up all the video, freezes and detection files, in case the external HDD is defective. For the HDD decryption, a service which decrypts and mounts these HDDs on boot was installed. To secure this data during run-time, a user account with limited permissions was created and is used for the auto startup. Therefore, this user can’t access the data on the system and only another account with administrator privileges may handle the sensitive data.

EndoMind software including a detailed installation handbook is freely available for research purposes:

<https://www.ukw.de/research/inexen/ai-applied-in-real-time/>

**Supplementary Table 1:**  **EndoMind hardware components.** Based on manufacturer's suggested retail price from April 2022.

| **Component** | **Device model** | **Price (€)** |
| --- | --- | --- |
| Grabber Card | DeckLink Mini Recorder 4K | 209 |
| Graphic Card | GeForce RTX 3080 Ti MSI Gaming Trio | 1449.90 |
| Motherboard | Gigabyte B550 VISION D | 258 |
| CPU | AMD Ryzen 7 3800X with Wraith PRISM | 299.90 |
| RAM | G.Skill RipJaws V F4-3200C16D-16GVKB | 66.50 |
| System Storage | Samsung 970 EVO Plus NVMe M.2 SSD | 89.90 |
| Record Storage | Western Digital Elements Portable | 122.90 |
| Power Supply | Antec HCG750 80+ Gold | 124.90 |
| Case | Corsair Carbide 275R | 109.90 |
| Galvanic isolator | LEN LHDGI01 Video isolator | 150.48 |

**Supplementary Table 2: Usability survey.** Items 1 to 10 are adapted from the System Usability Scale. Likert scale ranges from 1 (strongly disagree) to 5 (strongly agree).

|  | **Question** | **Answer Type** |
| --- | --- | --- |
| 1 | I think that I would like to use EndoMind frequently. | Likert scale |
| 2 | I found EndoMind unnecessarily complex. | Likert scale |
| 3 | I thought EndoMind was easy to use. | Likert scale |
| 4 | I think that I would need the support of a technical person to be able to use EndoMind | Likert scale |
| 5 | I found the various functions in EndoMind were well integrated. | Likert scale |
| 6 | I thought there was too much inconsistency in the EndoMind. | Likert scale |
| 7 | I would imagine that most people would learn to use EndoMind very quickly. | Likert scale |
| 8 | I found EndoMind very cumbersome to use. | Likert scale |
| 9 | I felt very confident using EndoMind. | Likert scale |
| 10 | I needed to learn many things before I could get going with EndoMind. | Likert scale |
| 11 | Estimated percentage of polyps identified by EndoMind prior to examiner. | 0 - 100 |
| 12 | Estimated percentage of polyps not identified by EndoMind. | 0 - 100 |
| 13 | I see an overall benefit in applying EndoMind in screening colonoscopy. | Likert scale |
| 14 | I would implement EndoMind into daily clinical practice. | Likert scale |
| 15 | I found false positive detections as disturbing | Likert scale |

**Supplementary Table 3: Examiner characteristics (n = 4).**

| **Characteristic** | **Value** |
| --- | --- |
| Age, mean (min., max.) | 60.7 (55, 65) |
| Gender |  |
| Female | 0% |
| Male | 100% |
| Type of center |  |
| Hospital | 1 |
| Outpatient practice | 3 |
| Years performing colonoscopies, mean (min, max) | 28.5 (23, 36) |
| Performed colonoscopies (total) |  |
| <500 | 0 |
| 500-2000 | 0 |
| 2000-10000 | 0 |
| >10000 | 4 |
| Performed colonoscopies (last 12 months) |  |
| <500 | 0 |
| 500-1000 | 1 |
| 1000-1500 | 0 |
| 1500-2000 | 2 |
| 2000-2500 | 1 |
| >2500 | 0 |

**Supplementary Figure:**

**
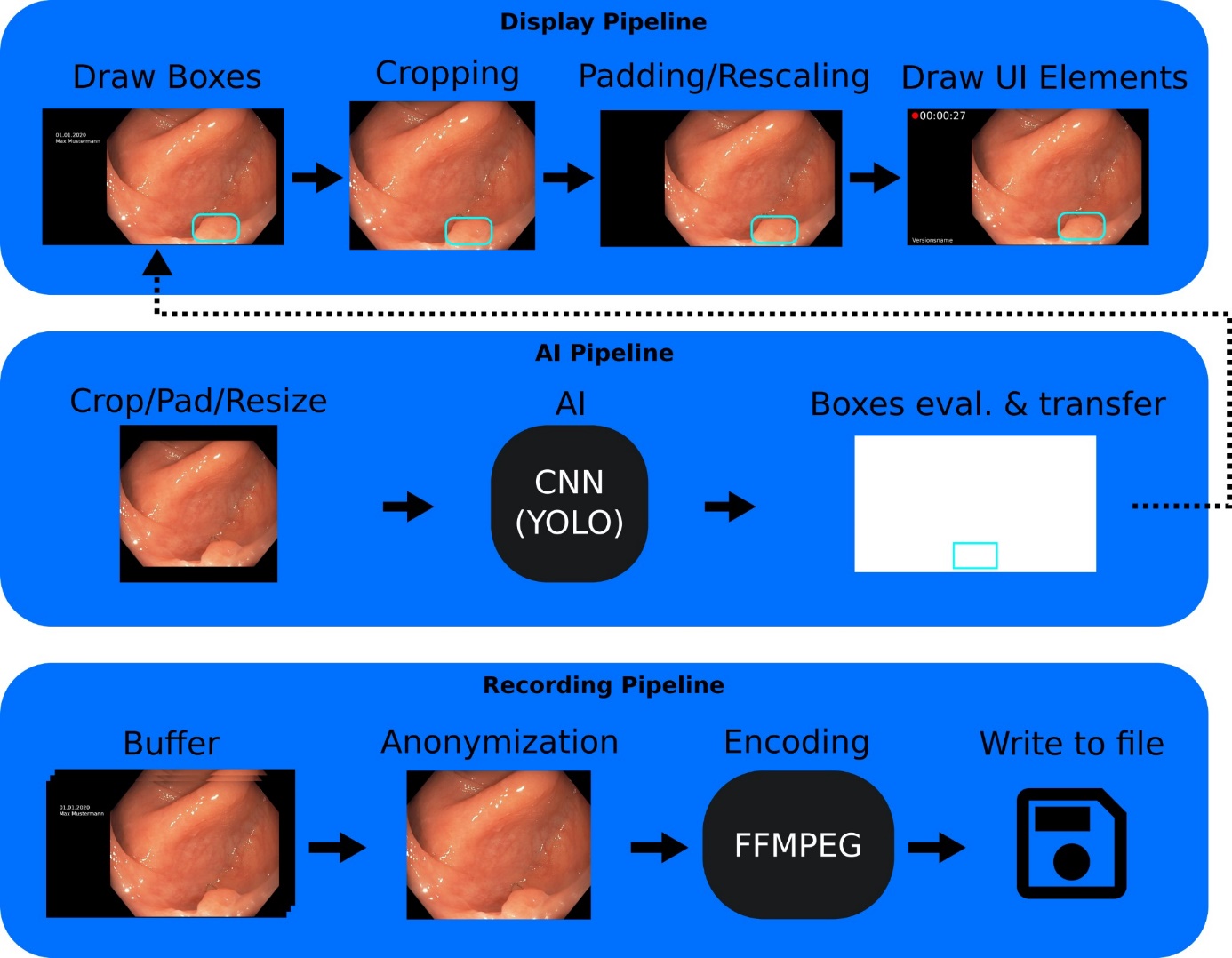
**

**Supplementary Figure 1;** **Flowchart describing the three independent core-processing pipelines of EndoMind**. To minimize delay of the endoscopy video, the display pipeline processes and visualizes the received images with detection boxes of the preceding frames. CNN, convolutional neural network; YOLO, you only look once; FFMPEG, fast forward motion picture experts group.

**Supplementary References:**

[1] Redmon J, Farhadi A. YOLOv3: An Incremental Improvement. ArXiv180402767 Cs 2018;
